# Intra-articular Osteoid Osteoma of the Olecranon Fossa

**DOI:** 10.7759/cureus.27484

**Published:** 2022-07-30

**Authors:** Corey K Ho, Jacob Azurdia, Andrew Park, Michael R Clay, David Gimarc

**Affiliations:** 1 Department of Radiology, University of Colorado Anschutz Medical Campus, Aurora, USA; 2 Department of Orthopedics, University of Colorado Anschutz Medical Campus, Aurora, USA; 3 Department of Pathology, University of Colorado Anschutz Medical Campus, Aurora, USA

**Keywords:** primary bone lesion, joint pain, intra-articular mass, benign bone tumor, osteoid osteoma

## Abstract

Osteoid osteomas are benign primary bone lesions characterized by a central nidus with surrounding reactive sclerosis, classically presenting as worsening nocturnal pain relieved by non-steroidal anti-inflammatory medications (NSAIDs). These most commonly occur in intracortical bone and the diaphysis of long bones. As a rare entity, intra-articular osteoid osteomas present unusually, often resulting in a delayed or incorrect diagnosis. We present a case of an intra-articular osteoid osteoma, emphasizing the importance of MRI in aiding diagnosis in this atypical location.

## Introduction

Osteoid osteomas are benign primary bone lesions characterized radiographically by the presence of a central nidus with surrounding sclerotic bone on plain radiographs [1]. An osteoid osteoma most commonly occurs in the first three decades of life and has a strong male predilection [2,3]. The tumor classically presents with an insidious onset of pain that is worse at night and relieved by non-steroidal anti-inflammatory medications (NSAIDs). The postulated mechanism by which this lesion causes pain is due to vasodilation-related stimulation of unmyelinated nerve endings within the nidus secondary to significant lesional prostaglandin synthesis [3,4]. This increased prostaglandin production also explains the sensitivity of this lesion to NSAIDs [5].

Osteoid osteomas are commonly found in intracortical bone and the diaphyses of long bones [1]; however, the intra-articular variety is an unusual presentation that often results in a delayed or incorrect diagnosis. Inappropriate treatment can lead to joint contracture and decreased range of motion [1]. This case report of an intra-articular osteoid osteoma of the olecranon fossa adds to the existing literature describing cases of osteoid osteomas in this atypical location. Our case report also underscores the importance of magnetic resonance imaging (MRI) when radiographs and computerized tomography (CT) do not readily aid in diagnosis.

## Case presentation

A 24-year-old male presented with two years of intermittent, gradual onset, deep left elbow pain. Conservative treatments, including physical therapy and NSAIDs, were minimally helpful. Over time, significant pain with elbow extension resulted in the development of a flexion contracture with an elbow range of motion of 15-100 degrees. Supination and pronation were preserved.

**Figure 1:**
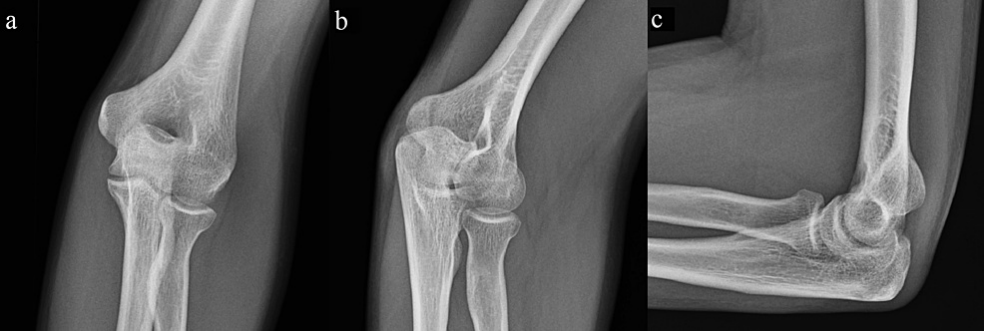
Preoperative radiographs of the left elbow Anteroposterior (a), oblique (b), and lateral (c) radiographs of the left elbow demonstrate no visible abnormality.

**Figure 2:**
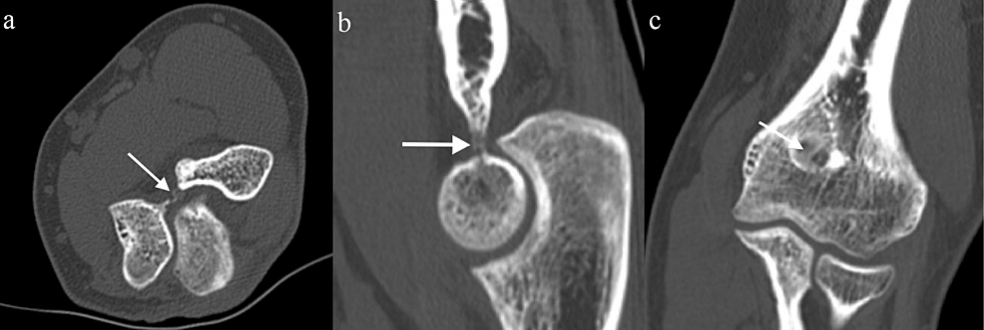
Preoperative CT scan of the left elbow Axial (a) and sagittal (b) images show a sub-centimeter lucent lesion with central sclerotic focus (arrow) within the bony margin of the olecranon fossa. A small amount of adjacent sclerotic reactive bone is seen on the coronal (c) images (arrow).

**Figure 3:**
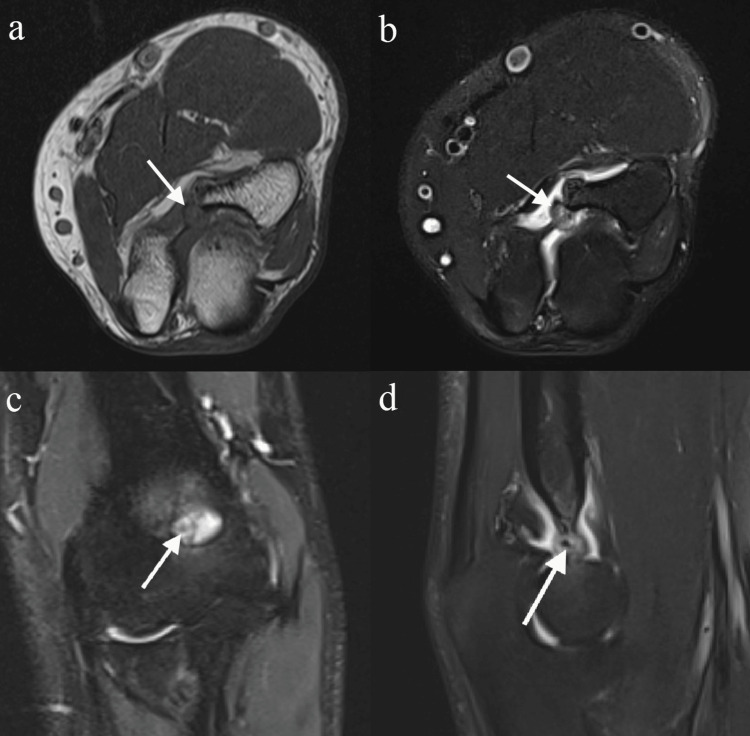
Preoperative MRI of the left elbow Axial T1 (a) and axial T2 fat-saturated (b) images demonstrate the expansile hypointense T1 and mixed hypointense/hyperintense T2 lesion arising from the olecranon fossa with thin cortical rim (arrow). Coronal T2 fat-saturated image (c) shows the central nidus (arrow) within the lesion surrounded by marrow edema. Sagittal T2 fat-saturated image (d) depicts a small hypointense nidus within the lesion (arrow) and a small effusion with some synovitis.

Accompanying the patient were radiographs and a CT scan of the left elbow, obtained one year prior. The radiographs demonstrated no significant abnormality (Figure 1). Retrospectively, the CT scan demonstrated a sub-centimeter lucent expansile lesion within the olecranon fossa (Figure 2). He underwent a subsequent MRI examination of the left elbow, which showed an expansile lesion arising from the olecranon fossa with surrounding marrow edema (Figure 3).

**Figure 4:**
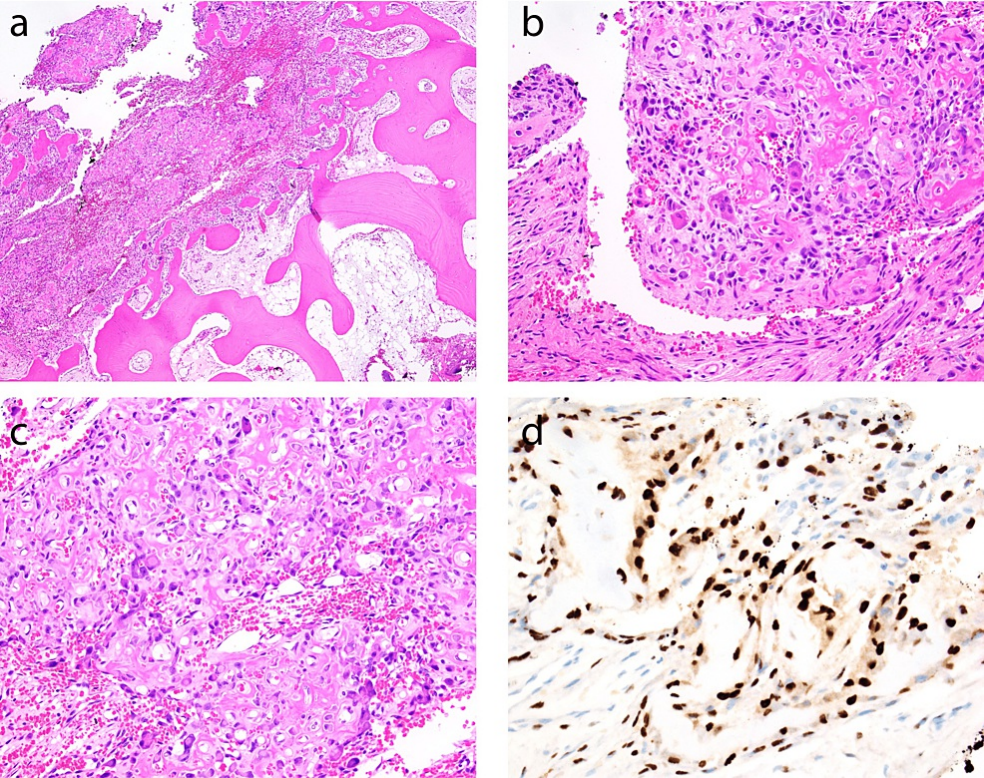
Histologic and immunohistochemical studies Hematoxylin and eosin (HE) stained section seen at 20× magnification (a) highlights a sudden transition point between background bone/marrow (bottom right) and a cellular lesion (top left). Higher power magnification of HE stained sections (b,c) reveals an osteoblastic process comprised of epithelioid osteoblasts with eccentric, brightly eosinophilic cytoplasm admixed with delicate osteoid matrix and osteoclastic giant cells. No cytologic atypia is identified and the background stroma is highly vascularized. SATB2 immunohistochemical staining (d) highlights osteoblasts rimming collections of disorganized osteoid material.

En-bloc resection of the olecranon fossa via the Outerbridge-Kashiwagi procedure [6] was performed. Histologic analysis revealed a hypercellular lesion comprised of epithelioid osteoblasts lining spicules of reactive appearing woven bone. Special AT-rich binding protein 2 (SATB2) immunohistochemical staining highlighted osteoblasts rimming collections of disorganized osteoid material. These findings were consistent with osteoid osteoma (Figure 4). Two years following the procedure, the patient reported a satisfactory outcome with complete resolution of pain and fully recovered range of motion of the elbow.

## Discussion

Osteoid osteomas occur within the femur or tibia in 50% of cases, usually at the metaphyseal-diaphyseal junction [2,4]. However, presentation in nearly every bone has been reported [7]. Intra-articular location is rare and comprises only 10% of cases [8], most of which involve the hip [9]. Intra-articular osteoid osteomas can be especially tricky to diagnose due to their clinically nonspecific presentation, which often includes arthralgias, stiffness, and swelling due to joint effusion [10]. The prostaglandins often elicit synovitis as they can freely flow within the joint [1]. Osteoid osteoma of the elbow, in particular, has been reported to present as a monoarthritis with joint effusion, rather than the typical nocturnal pain responsive to NSAIDs [11,12]. Unsurprisingly, literature on this topic has described confusion of this entity with a variety of diagnoses such as lateral epicondylitis, osteochondral defect, hemophilic arthropathy, infection, and trauma [2-5,8,13]. When present in the elbow, pain can limit flexion and extension, with sparing of supination and pronation. This pattern of pain, swelling, and muscle atrophy is often present along with the inconsistent response to NSAID treatment as previously described [3].

In the elbow, the most common location for intra-articular osteoid osteoma is in the distal humeral epiphysis [3]. Diagnosis, regardless of location, is dependent upon imaging using plain radiography, CT, MRI, and sometimes nuclear medicine bone scan [4]. Typical radiographic findings of osteoid osteoma are much more difficult to identify when the location is intra-articular as there is a significant overlap of bony margins. The reactive bone may be mistaken for a normal cortex. The nidus may also be small and difficult to visualize, especially in the absence of reactive bone [14]. This is thought to be due to the lack of cambium-containing progenitor cells in osteoblasts [15].

While CT has historically been touted as having a higher sensitivity and specificity in the diagnosis of osteoid osteoma when compared to MRI, recent studies now consider MRI to be equal to or even slightly better at detecting the nidus [9]. This is especially true of osteoid osteoma in atypical locations, in which dynamic MRI was shown to outperform nonenhanced MR and CT in the detection of the nidus [16]. As was seen in our case, imaging by CT alone may result in missed findings and, ultimately, misdiagnosis or delayed diagnosis. It has been reported that the time to diagnosis for intra-articular osteoid osteoma is 26.6 months, compared to about nine months for other locations [7]. In our case, the time to diagnosis was 24 months, at which point the patient had already developed a flexion contracture at the elbow. It was only after the patient obtained an MRI that the diagnosis was made, with findings revealing the intra-articular, expansile osteoid osteoma within the olecranon. A small effusion and synovitis were also present, associated with findings that have been previously reported [17].

Pathologic analysis characteristic of osteoid osteoma reveals the usual gross appearance of a round or oval central nidus with hyperemic features. Microscopically, the nidus appears as an immature woven bone with variable mineralization and interconnecting trabeculae, often with scattered osteoclasts. These specimens are small in size with a lack of pleomorphism that is well circumscribed [18]. As an immunohistochemical marker, SATB2 helps identify osteoblasts and reactive bone formation [19].

Following diagnosis by MRI, the patient underwent open excision of the lesion. Though ablation is typically the treatment of choice in cases of elbow osteoid osteoma, open excision with adhesiolysis has been described as an alternate therapy that is particularly helpful in the restoration of motion [20]. Consistent with the literature, the patient experienced a dramatic improvement in pain and range of motion following excision, with the recovery of a full range of motion following recovery from the procedure.

## Conclusions

Intra-articular osteoid osteomas rarely occur within joints. Proper identification of these often-small lesions is essential to guiding treatment. This case highlights the difficulty in diagnosing a rare intra-articular osteoid osteoma of the elbow and the increased patient morbidity that may occur as a result of diagnostic delay. While radiographs and CT are often regarded as the best imaging modalities for diagnosing this entity, our case serves to emphasize the importance of MRI in atypical cases where radiographs and CT are equivocal.
